# Serine/Arginine Protein Kinase Inhibitors Potentiate Melanoma Cell Death and Metastatic Inhibition Through Apoptotic Protein Triggering and Vimentin Dysregulation

**DOI:** 10.34172/apb.025.43291

**Published:** 2025-07-19

**Authors:** Atit Silsirivanit, Chaturong Inpad, Jesadagorn Siriwath, Sittiruk Roytrakul, Sukanya Horpaopan, Kanyanut Kotanart, Worasak Kaewkong

**Affiliations:** ^1^Department of Biochemistry, Faculty of Medicine, Khon Kaen University, Khon Kaen 40002, Thailand; ^2^Department of Biochemistry, Faculty of Medical Science, Naresuan University, Phitsanulok 65000, Thailand; ^3^Department of Optometry, Faculty of Allied Health Science, Naresuan University, Phitsanulok 65000, Thailand; ^4^National Center for Genetic Engineering and Biotecnology (BIOTEC), Pathumthani, 12120, Thailand; ^5^Department of Anatomy, Faculty of Medicine, Chiang Mai University, Chiang Mai 50200, Thailand

**Keywords:** Apoptosis, Melanoma, Metastasis, SRPK

## Abstract

**Purpose:**

Melanoma arises from the malignant transformation of melanocytes, a serious health problem in high UV-exposure countries. Ineffective treatments and metastasis have led to poor prognosis and high mortality among melanoma patients. Several underlying mechanisms are suspected. The splicing-error in many genes has been frequently reported in melanoma, therefore, targeting splicing regulator Serine/Arginine protein kinases (SRPKs) is promising.

**Methods:**

SRPKs expression in the TCGA dataset was analyzed by GEPIA. A375 and MNT-1 were comparatively treated by SRPK inhibitors, SRPIN340 and SPHINX31. Effects on viability and growth were measured by MTT and hanging drop assay. Apoptotic death was examined by flow cytometry and western blotting. Invasive ability was determined by transwell assay. Invasive-associated genes, proteins, and enzymes were tracked by RT-PCR, western blotting, immunofluorescence, and gelatin zymography.

**Results:**

SRPIN340 exhibited higher inhibitory effects on the viability and growth of melanoma cells than SPHINX31. Apoptotic induction was found with downregulated Bcl-2 and upregulated cytochrome c, especially in A375 cells. For metastatic inhibition in A375, lower numbers of invaded cells were counted. Downregulated vimentin mRNA and transcription factors-snail, as well as altered vimentin protein expression and localization were marked. Remarkably, the activities of MMP2 and MMP9 were suppressed.

**Conclusion:**

SRPK inhibitors potentially suppressed melanoma cell survivability and metastasis through the triggering of apoptotic proteins and dysregulating vimentin. These collected data serve as a basis for utilizing new alternative therapeutic strategies by targeting splicing regulator SRPK, for melanoma treatment.

## Introduction

 Melanomas occur in the form of malignant tumors arising from the abnormal growth and transformation of melanocytes which can be found in various anatomical locations, especially on the body surfaces. Melanomas can develop anywhere on the human body, but most often advance in areas that have had exposure to ultraviolet (UV) light from the sun, such as the back, arms, and face. Melanoma is classified based on the tissue origins, and the most common type is cutaneous melanoma under the skin layer. Increasing incidence of melanoma is reported in North American, Australasian, and European populations.^1^ The important risk factors of melanoma are UV radiation and genetic mutation of Serine/Threonine-protein kinase B-Raf (BRAF) into the BRAFV600E variant.^1,2^ In the countries with high UV-exposure including Southeastern Asia, particularly Thailand, melanoma is also recorded as the cause of cancer mortality.^3^ There are various guidelines and strategies to shape precise melanoma therapies, but these still remain ineffective or reveal resistant phenomena.^4-6^ Remarkably, the metastasis of melanoma is the lethal type of this cancer, as the most highly mutated, heterogenous, and aggressive.^7^

 A number of studies reported the translational impact of oncogenic mRNA transcripts/variants derived from aberrant alternative splicing in cancers, including gene splicing errors to get protein isoforms with oncogenic properties, upregulation of splicing factors, or dysregulation of splicing regulators. Therefore, this evidence suggested the addition of aberrant alternative splicing as another hallmark of cancer.^8^ The molecular basis of this post-transcriptional modification results in a multi-exon gene being coded for multiple mRNA transcripts, then they can be translatable into various protein isoforms.^9^ Aberrant alternative mRNA splicing contributes to the cancer development including lung, liver, and breast cancer.^10-12^ In a recent study in 2023, 174 melanoma patients from tertiary hospitals in Thailand with pathologically confirmed conditions were pronounced to have a 5-year overall survival (OS) rate of 43%, and the median survival period was only 3.91 years. Palpable lymph nodes, distant metastases, and lympho-vascular invasion were also marked.^13^

 The cellular mechanism of alternative splicing is regulated by a family of serine/arginine-rich splicing factors (SRSFs). SRSFs play a crucial role in the spliceosome machinery to process pre-mRNA into mature transcripts. Twelve members of SRSFs contain one or two domains of RNA recognition motifs and different lengths of arginine-serine rich domains. SRSF1 to SRSF12 have their specific binding region with distinct 6–10 nucleotide sequences.^14^ Therefore, dysregulation or overexpression of SRSFs can enable the events of splicing errors, and then the aberrant transcripts might be translatable into cancer related-proteins gaining oncogenic properties and potentially facilitating cancer progression.^15^

 Phosphorylation of SRSFs by serine-arginine protein kinases (SRPKs) is the key approach to regulate splicing processes. The oncogenic functions of SRPKs were demonstrated in many cancer models, by regulating the alternative splices of tumor suppressor genes into opposite functions, and on the other hand enabling oncogene splicing to enhance cancer aggressiveness.^16-18^ Of note, once SRPKs became evident as the target for cancer treatment, the specific chemical substances to block or suppress SRPK functions were developed, from the first pan-SRPK inhibitor SRPIN340,^17^ to the structural modified version as SRPK1-specific inhibitor, SPHINX31.^19^ Several studies demonstrated the targeting of SRPKs and examined the functional involvement both *in vitro* and *in vivo* by gene silencing and gene overexpression, as well as SRPK inhibitors.^16,18^ Remarkably, the SRPK inhibitors considered for this approach include SRPIN340, which has previously demonstrated *in vivo *efficacy in melanoma,^16^ and SPHINX31, developed specifically for targeting SRPK1, a kinase implicated in the upregulation observed in melanoma tissues. This study aims to determine the clinical relevance of SRPK1 and SRPK2 expression regarding dataset analysis before examining the effects of SRPK inhibitors, SRPIN340 and SPHINX31, on cell viability and growth in the melanoma cell lines, A375 and MNT-1. Subsequently, the inhibitory effects on melanoma cell survivability including apoptotic induction and activation of apoptotic pathway can be examined. In addition, the inhibition of the metastatic associated phenotype, especially invasion ability, as well as the deactivation of vimentin expression and MMP2/MMP9 inactivation can be measured.

## Materials and Methods

###  Dataset analysis of target gene transcripts

 The on-web analytic tool, GEPIA (http://gepia.cancer-pku.cn/) based on TCGA and GTEx datasets was used. Box-plot analysis was used to describe the expression changes (transcripts per million, TPM) of SRPK1 and SRPK2 between tumor (T) and normal (N) tissues. Survival analysis was used to represent the relationship between SRPK1 or SRPK2 expression and OS or disease-free survival (DFS).

###  Cell lines and cell culture

 HaCaT (human keratinocyte cell line), A375 (human cutaneous melanoma cell line) and MNT-1 (highly pigmented human melanoma cell line) were used in this study.Dulbecco’s Modified Eagle Medium (DMEM, Gibco, Thermo Fisher Scientific, MA, USA)supplemented with 10% (v/v)fetal bovine serum (FBS, Gibco)and 100 units/mL ofAntibiotic-Antimycotic (Gibco) was used as culture medium. Cultured cells were maintained at 37°Cunder 5% CO_2_ and sub-passage twice aweek using 0.025% (w/v) trypsin/EDTA (Gibco).

###  SRPK inhibitors

 SRPIN340 and SPHINX31 were purchased commercially from the Cayman Chemical Company (MI, USA). Dimethylsulfoxide (DMSO, Sigma-Aldrich, MO, USA) was recommended as an appropriate diluent for preparing as stock solution and working concentrations, therefore, 0.5% (v/v) DMSO treated cells was added for vehicle control for all treatment conditions.

###  Cell viability assay

 The cultured cells were plated in a 96-well plate at 1 × 10^4^ cells per well for 24 h to represent the comparable confluency of adhered cells. After 24, 48, and 72 h treatment by SRPK inhibitors or vehicle control, 100 μL of 3-(4,5-dimethylthiazol-2-yl)-2,5-Diphenyltetrazolium Bromide (MTT, Biobasic) was added to each well, follow-on a final concentration of 0.5 mg/mL (in triplicate for each treatment condition). After 4 h incubation, the MTT-containing medium was removed, and the formazan crystals were dissolved in DMSO. Finally, the absorbance was measured colorimetrically at 540 nm using a Synergy HT Multi-Detection Microplate Reader (BioTek Instruments, Inc., Winooski, VT, USA).

###  Cell spheroid formation by hanging drop assay

 After 24 h treatment by SRPK inhibitors or vehicle control, 5 × 10^3^ cells in 10 μL of treated cell suspensions were hanging dropped on the lids of the cell culture dishes (10 drops per condition) and incubated at 37 °C in 5% CO_2_. Then, the formations of cell spheroids were observed and imaged over an inverted microscope at 1, 3, 5, and 7 days.

###  Cell apoptosis by flow cytometry

 Annexin V-Phosphatidylethanolamine (PE)/7-Amino-Actinomycin (7-AAD) staining was used for the determination of apoptotic cell populations.The cultures were plated in a 6-well plate at 2.5 × 10^5^ cells per well for 24 h to represent the comparable confluency of adhered cells. After 24 h treatment by SRPK inhibitors or vehicle control, 100 μL of Muse^TM^ Annexin V & Dead Cell reagent (Cytek Biosciences, Fremont, CA, USA) and an equal volume of 4 × 10^5^ cells from each treatment groups were mixed. After 20 min incubation at room temperature, the numbers of live, dead and apoptotic cells were analysed using Muse^®^ Cell Analyzer and Muse 1.5.0.0 Analysis software (Merck KGaA, Darmstadt, Germany).

###  Protein extraction, concentration measurement, and western blot analysis

 The protein samples of whole cell lysate from each of the treatment groups were isolated using a radioimmunoprecipitation assay buffer (RIPA, Thermo Fisher Scientific). The Bradford assay was used for concentration measurement by mixing 200 μL of Bradford solution (Bio-Rad laboratories, Hercules, CA, USA) with 1 μL of extracted proteins with 10-fold dilution, or Bovine serum albumin (BSA, Capricorn scientific, Ebsdorfergrund, Germany) at 0, 0.1, 0.2, 0.3, 0.4, and 0.5 mg/mL, which was arranged as standard proteins to calculate as the standard curve. After 4 h of incubation, the absorbance was measured at 595 nm for calculating the protein concentration. Equal amounts of protein from each of the treatment groups were separated in SDS-PAGE gel (5% stacking gel and 12% separating gel), transferred to PVDF membranes (Bio-Rad Laboratories), and blocked by 5% BSA. The blotted membrane was probed with primary antibodies: rabbit anti-human Bcl-2 (1:5,000, Elabscience, Houston, TX, USA), rabbit anti-human cytochrome c (1: 5,000, Cell signaling Technology, Danvers, MA, USA), rabbit anti-human vimentin (1: 2000, Merck KGaA) and rabbit anti-human GAPDH (1: 10,000, Merck KGaA) at 4 °C, and left overnight before subsequently being probed with secondary antibodies: 1:10,000 of HRP-conjugated goat-anti-rabbit IgG (Merck KGaA) or goat-anti-mouse IgG (Merck KGaA) for 1 h. The protein bands were detected with Immobilon® ECL Ultra Western HRP Substrate (Merck KGaA), imaged by Amersham ImageQuantÔ 800 biomolecular imager (Cytiva, Amersham, UK). Lastly, the signal intensity of each band was quantified by ImageJ software as a semi-quantitative expression which was normalized by GAPDH protein intensity.

###  Transwell cell invasion assay

 The cell invasion ability of each treatment group was comparatively evaluated using a Boyden chamber assay. The transwells (polycarbonate membrane) with 8 µm-pore size were coated by 0.4 mg/mL Matrigel matrix (Corning, Corning, NY, USA) and inserted as the upper chamber. In the lower chamber, 600 µL of complete media was added for serving as the enrichment layer. The 2 × 10^4^ cells of each treatment group were seeded into the upper chambers with 100 µL of serum free media. A total of 16 h was allowed for vertical invading, and the invaded cells at the bottom of the upper chamber were fixed with 4% paraformaldehyde and stained with 4% crystal violet for subsequent imaging and counting.

###  Preparation of RNA and reverse transcription-polymerase chain reaction (RT-PCR)

 The RNA samples were isolated from harvested cells from each treatment group using the E.Z.N.A^®^ Total RNA kit I (Omega Bio-Tek, Norcross, GA, USA). RNA concentration was measured for preparing into 1 μg for complementary DNA (cDNA) synthesis using iScript^TM^ Reverse Transcription Supermix (Bio-Rad Laboratories). To determine the gene expression, PCR was performed by preparing the 20 µL-reaction mixture with 200 ng cDNA, 0.4 µM of each of the forward and reverse primers and 1X MyTaq^TM^ HS Red Mix (Bioline Reagents Limited, London, UK). The sequences of amplification primers for Vimentin, Snail, and GAPDH are listed in Table 1. The thermocycling conditions were 95˚C for 5 min pre-denature, followed by 30 cycles of 95˚C for 30 s, 55 ˚C for 30 sec and 72 ˚C for 30 s, before 72 ˚C for 5 min as the final extension. PCR products were mixed with 6X fluorescent DNA staining reagent (Novel Juice, Gene DireX, Taoyuan, Taiwan), analyzed by 2% agarose gel electrophoresis at 90 volts for 30 min, detected by the Amersham ImageQuantÔ 800 biomolecular imager (Cytiva) and quantified using ImageQuant TL 7.0 software (GE Healthcare Life Science, Amersham, UK) in which GAPDH intensity was used for semi-quantitative normalization.

**Table 1:** Sequences of specific primers used in this study.

**Primer name and product size (bp)**	**Sequence (5'->3')**	**Bases**
Vimentin_213	Forward primer: TCT ACG AGG AGG AGA TGC GG	20
	Reverse primer: GGT CAA GAC GTG CCA GAG AC	20
Snail_231	Forward primer: ACC ACT ATG CCG CGC TCT T	19
	Reverse primer: GGT CGT AGG GCT GCT GGA A	19
GAPDH_174	Forward primer: TTG CCA TCA ATG ACC CCT TCA	21
	Reverse primer: CGC CCC ACT TGA TTT TGG A	19

###  Immunofluorescence staining

 The 1.6 × 10^4^ cells of each treatment group were cultured in 8-well culture slides for 24 h. Then, the adhered cells were fixed by 4% paraformaldehyde (Merck KGaA), permeabilized by 0.2% Triton X-100 (Merck KGaA), and blocked for non-specific binding by 5% FBS. Then, the cells were incubated with rabbit anti-human vimentin (1: 500) at 4˚C, overnight. The reactivity was detected via incubation with FITC-conjugated goat-anti-rabbit antibody (1: 200) for 1 h at room temperature. Nuclei were stained by 1: 10,000 DAPI (Merck KGaA) and the fluorescence signal was observed under the Nikon fluorescence microscope (Nikon Corporation, Tokyo, Japan).

###  Gelatinase activity assay by gelatin zymography

 The activities of the matrix metalloproteinases, MMP2 and MMP9, were evaluated by gelatin zymography assay. The culture medium (conditioned medium) from each treatment group was collected. The protein concentration was measured using the Bradford assay for preparing 0.6 µg to load into 10% SDS-PAGE gels with 0.01% (w/v) gelatin (Merck KGaA). The gels were incubated in developing buffer with CaCl_2_ and ZnCl_2_ overnight, and subsequently stained with 0.5% Coomassie brilliant blue R-250 (Sigma-Aldrich) until clear bands (gelatin digestion) were observed. The density of the clear bands which specified kDa was calculated as gelatinase activities.

###  Statistical analyses 

 The data were presented as mean ± SD of independent experiments. Analyses were performed using Microsoft Excel (Microsoft Office Software, Redmond, WA, USA), GraphPad Prism version 8.0 (GraphPad Software Inc., San Diego, CA, USA), and ImageJ version 1.54 (National Institutes of Health, Bethesda, MD, USA). Statistical analyses were performed using Student’s t test for assessing the differences in continuous data. A *P *value less than 0.05 was considered to be significant, for which *,^#,$^*P* < 0.05, **,^##,$$^*P* < 0.01 and ***,^###,$$$^*P* < 0.001.

## Results

###  The transcript quantification of SRPK1 and SRPK2 genes in skin cutaneous melanoma (SKCM) from GEPIA analyses

**Figure 1:**
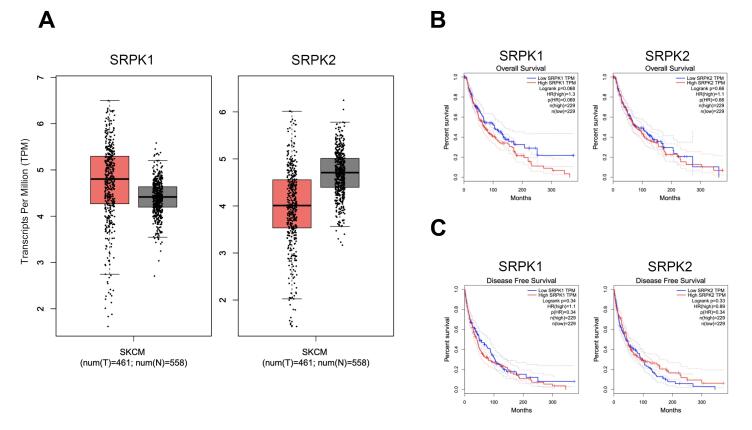
mRNA expression levels of SRPK1 and SRPK2 genes in skin cutaneous melanoma (SKCM) obtained from GEPIA analyses. **(A)** Transcripts per million (TPM) counts in 461 tumors and 558 non-tumor adjacent SKCM patients represented in a box-plot. **(B)** Association between SRPK1 and SRPK2 expression levels and overall survival (OS). **(C)** Association between SRPK1 and SRPK2 expression levels and disease-free survival (DFS)

 Comparatively, the transcripts per million (TPM) counts among 461 tumors (T) and 558 non-tumor adjacent (N) in melanoma patients were represented in a box-plot. The increasing number of SRPK1 transcripts accounted for the higher average in tumors when compared with non-tumor adjacent (Figure 1A). On the other hand, the decreasing trend of SRPK2 transcripts into a lower average in tumors was found. Remarkably, the association between SRPK1 and SRPK2 expression levels (TPM count) and overall and/or DFS were examined. From 461 tumors which were divided into 229 high vs. 229 low SRPK1 TPM, the decreasing OS was significant in high SRPK1 but not SRPK2 (Figure 1B) and a similar trend of analyses was considerable for DFS (Figure 1C).

###  The inhibitory effects of SRPIN340 and SPHINX31 on the viability and growth of A375 and MNT-1 melanoma cells

**Figure 2:**
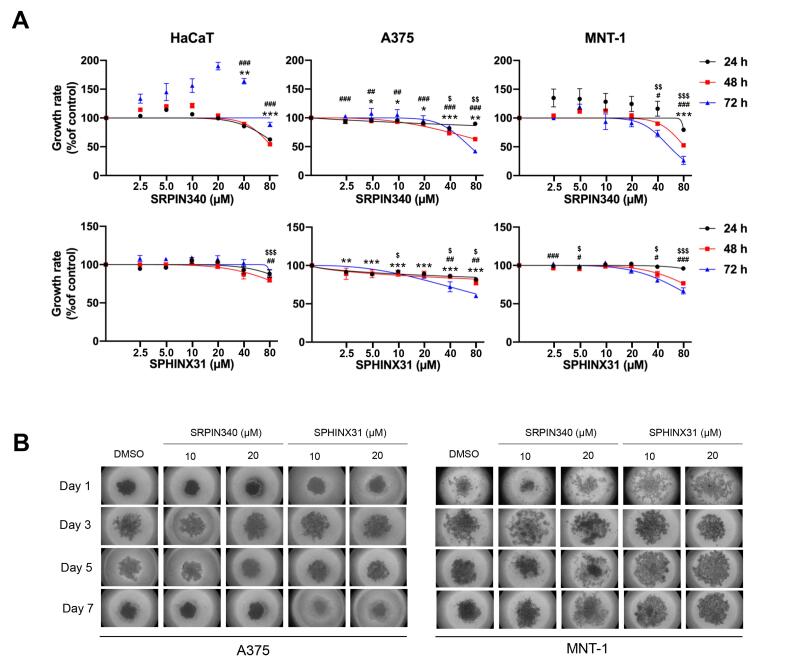
The inhibitory effects of SRPIN340 and SPHINX31 on cell viability and growth ability in HaCaT keratinocyte cell, A375 and MNT-1 melanoma cells.** (A) **The inhibitory effects on cell viability at 24, 48, and 72 h were assessed by MTT assay. **(B)** The inhibitory effect on growth ability was monitored for 7 days by hanging drop assay. Significant differences became evident and marked in the graphs when cell viability decreased. *,^#,$^*P* < 0.05, **,^##,$$^*P* < 0.01 and ***,^###,$$$^*P* < 0.001, compared with the control group of each experiment: *control vs. 24 h, ^#^control vs. 48 h, ^$^control vs. 72 h

 For cell viability at the indicated time-points of 24, 48, and 72 h using 0, 2.5, 5, 10, 20, 40, and 80 µM of inhibitors, In A375 cells, cell viability decreased to 63.00% at 48 h and 41.97% at 72 h. In MNT-1 cells, viability decreased to 52.73% at 48 h and 26.34% at 72 h. The half-maximal inhibitory concentration (IC50) values of SRPIN340 were determined to be 70.34 µM in A375 cells and 55.81 µM in MNT-1 cells at 72 h. However, there was a decrease in cell viability once treated by SRPK1-specific inhibitor, SPHINX31, to 60.31% and 66.74% viability at 72 h in A375 and MNT-1, respectively. Remarkably, neither SRPIN340 nor SPHINX31 had no cytotoxic effect on inhibiting cell viability in keratinocytes (HaCaT) cell (Figure 2A). For the spheroid growth, the formation of spheroids was monitored every two days until 7 days using 0, 10, and 20 µM of inhibitors. SRPIN340 treatment showed the lower spheroid size under 20 µM after treatment for 3 and 5 days. Remarkably, the formation ability of the MNT-1 spheroid was lowered by both SRPIN340 and SPHINX31 as represented by loose aggregation of the treated cells, particularly in SRPIN340 treatment (Figure 2B).

###  The effects of SRPIN340 and SPHINX31 on the apoptotic induction of A375 and MNT-1 melanoma cells

**Figure 3:**
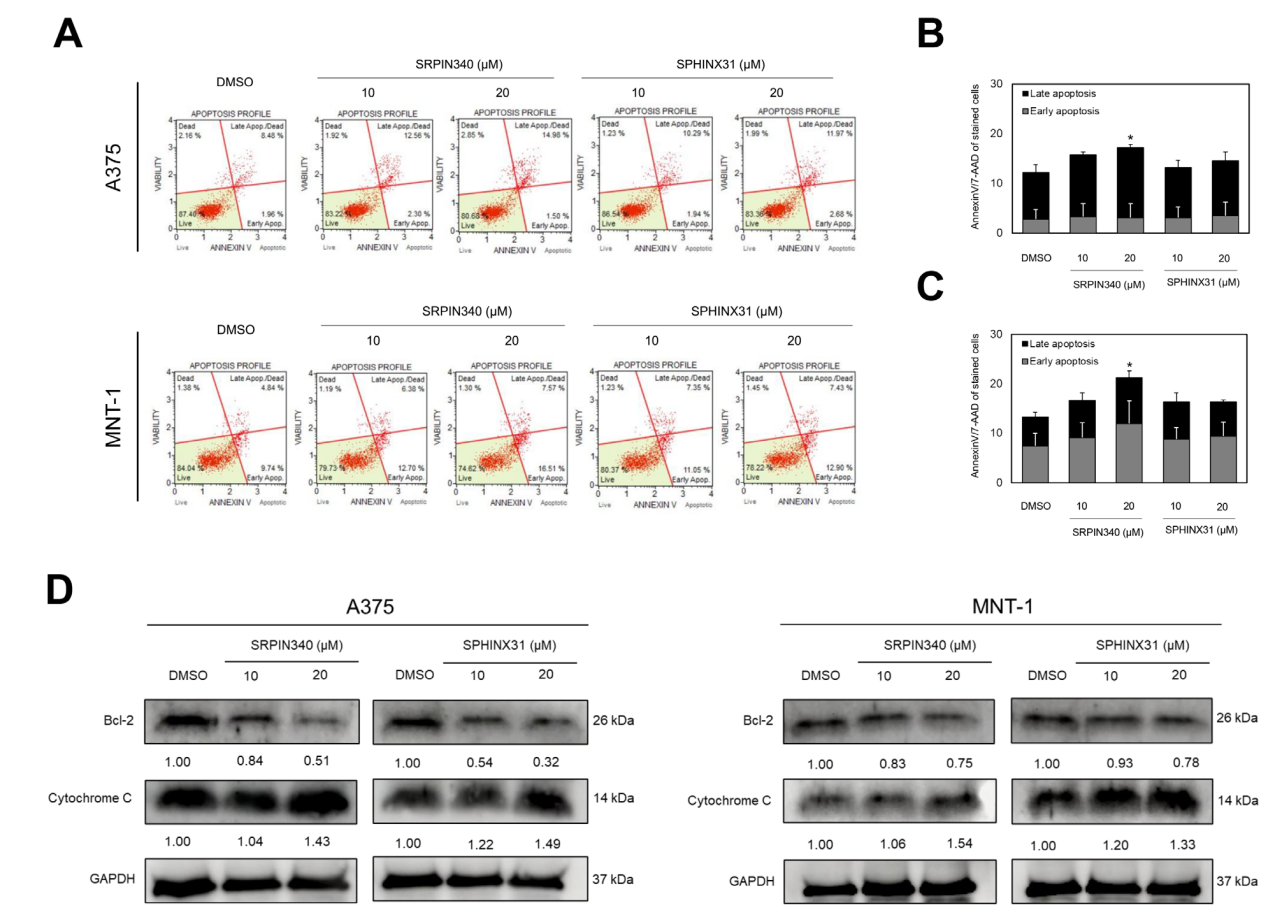
The effects of SRPIN340 and SPHINX31 on the apoptotic induction of A375 and MNT-1 melanoma cells.** (A) **Gating of early and late apoptotic cell population by AnnexinV/7-AAD staining, analyzed by flow cytometry. **(B)** The relative percentage of early and late apoptosis in A375. **(C)** The relative percentage of early and late apoptosis in MNT-1. **(D)** The expression of anti-apoptotic protein, Bcl-2 and pro-apoptotic protein, cytochrome c by western blotting analysis. *P < 0.05, **P < 0.01, and ***P < 0.001, compared with the control group of each experiment

 To examine the effects of SRPK inhibitors on cell death induction, dual staining of Annexin V/7-AAD was applied for gating the apoptotic cell populations using flow cytometry (Figure 3A). SRPIN340 demonstrated the higher apoptotic cell populations in a dose-dependent manner, when compared with SPHINX31. Curiously, SRPIN340 promoted the apoptotic cell induction in A375 into the late apoptotic phase (Figure 3B), whereas the apoptotic cell induction was promoted in MNT-1 into the early stage (Figure 3C). Moreover, the expression of apoptotic associated-proteins was validated. The anti-apoptotic protein Bcl-2 was downregulated as was the pro-apoptotic protein cytochrome c, and upregulated as in the dose-dependent manner of SRPIN340 and SPHINX31 treatment (Figure 3D).

###  The inhibitory effects of SRPIN340 and SPHINX31 on invasion ability through the expression of vimentin and MMP enzyme deactivation in A375 melanoma cells

**Figure 4:**
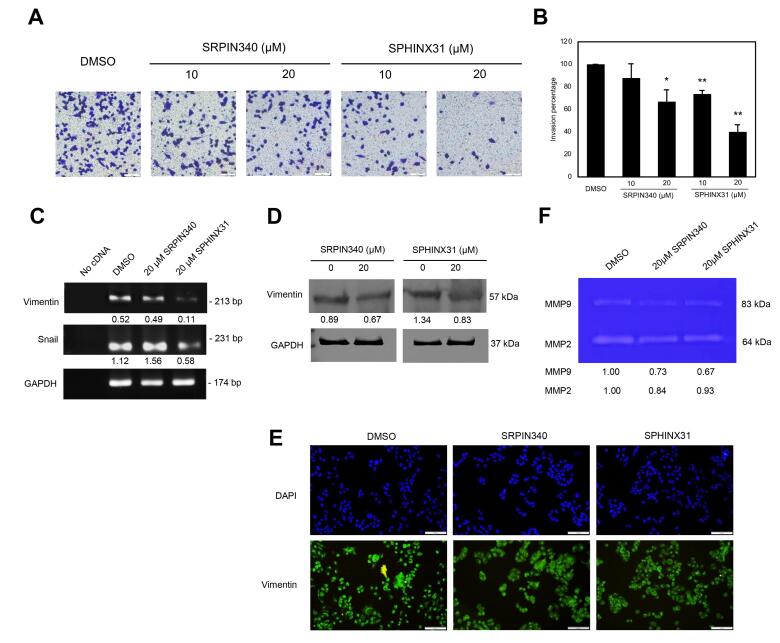
The inhibitory effects of SRPIN340 and SPHINX31 on invasion ability through the expression of vimentin and MMP enzyme deactivation in A375 melanoma cells.** (A) **Staining of invaded cells passed through the transwell invasion assay. **(B)** The relative percentage of invaded cells. **(C)** The mRNA expression of vimentin and the transcription factor-snail normalized with GAPDH mRNA by RT-PCR assay. **(D) **The expression of vimentin protein normalized with GAPDH protein by western blotting analysis. **(E) **The staining (localization and staining intensity) of cellular vimentin by immunofluorescence and cell nuclei marked by DAPI staining. **(F)** The gelatinase enzyme activity of MMP2 and MMP9 by gelatin zymography. *P<0.05, **P<0.01, and ***P<0.001, compared with the control group of each experiment

 For the metastatic-associated phenotype, a positive effect of the SRPK inhibitor to inhibit the invasion ability of A375 cells was observed. Distinct from previous results on viability, growth, and apoptotic induction, SPHINX31 significantly reduced the number of invaded cells in a dose-dependent manner, and represented a higher inhibitory effect than SRPIN340 (Figure 4A-B). The key morphological change of metastatic cell phenotype is epithelial-mesenchymal-transition (EMT), and the expression of vimentin was verified. Using RT-PCR, vimentin mRNA expression in A375 cells was greatly decreased by SPHINX31 treatment which was responsible for the downregulation of its transcription factors, snail mRNA (Figure 4C). Also, the downregulation of vimentin protein was validated by western blot analysis (Figure 4D). In addition, immunofluorescence presented the vimentin staining at lower intensity on A375 cells in both SRPK inhibitor-treated groups (Figure 4E). Moreover, SRPIN340 and SPHINX31 suppressed the activity of key invasive enzymes by decreasing the activities of cellular gelatinases MMP2 and MMP9 for catalyzing the gelatin substrate in gelatin zymography (Figure 4F).

## Discussion

 Melanoma is an enormously aggressive type of cancer, and a major problem in countries with extreme UV conditions, such as Thailand. The high mortality rate associated with its resistance to chemotherapy and its metastatic ability at secondary sites, including the lymph nodes, liver, lungs, brain, or bones, ensure high severity, poor prognosis, ineffective treatments, and shorter survival periods.^20^ The results of this study disclose the functions involved in gene-splicing dysregulation and how this contributes to cancer progression. It should be noted that the dysregulation of splicing factor protein kinases, SRPKs, both in expression and activation, potentially contributes to various diseases, especially the development of cancer,^15^ and crucially contributes to cancer cell aggressiveness.^21,22^ Therefore, SRPKs might be the key molecules which play an important role in tumor progression in terms of splicing regulator dysregulation.^23^

 Upregulation of SRPKs was publicized in many reports including studies in pancreatic cancer, breast cancer, colon cancer, prostate cancer, lung cancer, glioma, leukemia, and melanoma.^24-30^ SRPK1 upregulation is associated with poor prognosis of breast cancer,^31^ as well as with the metastasis-free and OS of lung cancer.^32^ This study showed the average transcript counts for which SRPK1was higher, but SRPK2 was lower in melanoma tumors when compared with the normal (Figure 1A). Correspondingly, the decreasing OS was significant in high SRPK1 patients, but not in high SRPK2 patients (Figure 1B). Therefore, SRPIN340 (an inhibitor of both SRPK1 and SRPK2) was selected based on strong *in vivo* evidence of its melanoma-suppressive effects,^16^ while SPHINX31 (an SRPK1-specific inhibitor) was chosen due to the increased count of SRPK1 transcripts (Figure 1A) and its clinical relevance to melanoma patient survival (Figure 1B-C).

 Several findings presented inhibition of SRPK1 using siRNA, shRNA and specific inhibitors on cell-based effects of cancers. The effect of the SRPK1/2 inhibitor, SRPIN340 was examined. In leukemia, the viability of various types of leukemia cell lines was suppressed in a dose-dependent manner once after 48 h of treatment by SRPIN340. Specifically, the IC50 values were determined as follows: K562 (52 μM), HL60 (44.7 μM), Nalm6 (66.6 μM), 697 (59.2 μM), Molt4 (92.2 μM), and Jurkat (82.3 μM).^17^ In melanoma, SRPK1 is involved in the angiogenesis of metastatic tumors, which presented the effect of SRPIN340 on cell cytotoxicity, reporting an IC50 value at concentrations above 100 μM and revealed that the tumor growth was attenuated after SRPIN340 treatment.^16^ Similar results were also reported in B16F10 murine melanoma cells for which the colony formation ability was reduced by SRPIN340 in dose-dependent treatment.^18^ Moreover, treatment by SPHINX31, the SRPK1-specific inhibitor, reduced the colony formation ability of A375 human melanoma cells both in size and number of colonies.^33^ In addition, SPHINX31 significantly suppressed the tumor growth of prostate cancer *in vivo*.^34^ This study also revealed that SRPIN340 showed significant inhibitory effects in melanoma cell lines, with IC50 values of 70.34 µM for A375 cells and 55.81 µM for MNT-1 cells at 72 h. Notably, both inhibitors had no inhibitory effect on the viability of HaCaT cells, indicating selective toxicity towards melanoma cells (Figure 2A). For the spheroid growth, SRPIN340 treatment showed the lower spheroid size under 20 µM after treatment for 3 and 5 days. Remarkably, the formation ability of MNT-1 spheroids was lowered by both SRPIN340 and SPHINX31, as represented by loose aggregation of the treated cells, particularly in SRPIN340 treatment (Figure 2B). On the other hand, to explore the death induction effects of SRPK inhibition, SRPIN340 and SPHINX31 increased the number of Annexin V-positive NK/T-cell lymphoma cells, which also marked the upregulation of cleaved PARP and cleaved caspase-3.^35^ In mice, SRPIN340 treatment in tumor melanoma increased caspase-3-positive cell counts.^36^ This study presented higher apoptotic cell populations as counted in SRPIN340 treatment than in SPHINX31 treatment (Figure 3A-C). Also, both SRPIN340 and SPHINX31 demonstrated dose-dependent effects in Bcl-2 downregulation and cytochrome c upregulation (Figure 3D). These observations are consistent with previous studies in cholangiocarcinoma, where the SRPK inhibitors SRPIN340 and SPHINX31, at 10 and 20 µM, induced a dose-dependent increase in early apoptosis. This was accompanied by mitochondrial leakage of cytochrome C and an elevated level of cleaved caspase-3 protein, indicating activation of apoptotic pathways.^37^

 The functional involvement of SRPKs in tumor metastasis has been recognized. A number of studies revealed the effect of SRPK inhibition on metastatic abilities using the scratched wound assay in A375 cells after the knockdown of SRPK1, and showed a reduction in migrating ability.^16^ In B16F10 murine melanoma cells, SRPIN340 treatment demonstrated inhibitory effects on migration, invasion, and adhesion abilities.^18^ In addition, SPHINX31 treatment showed significantly slower migration ability in A375 cells.^33^ In this study, suppression of the invasion ability of A375 cells using the transwell invasion assay was verified. SPHINX31 reduced the number of invaded cells in a dose-dependent manner, with higher inhibitory effects than SRPIN340 (Figure 4A-B).

 The key morphological changes of metastatic cell phenotype involve EMT. Therefore, the expression of key mesenchymal protein-vimentin was verified, and mRNA expression of vimentin and its transcription factor-snail in A375 cells was decreased by SPHINX31 treatment (Figure 4C). Moreover, vimentin protein expression and localization were also altered (Figure 4D-E). A previous study used microRNA targeting SRPK1, and overexpression of miR-1296 to evaluate the expression of N-cadherin and vimentin in hepatocellular carcinoma HCCLM3 cells.^38^ Overexpression of SRPK1 promoted cell invasion ability and the upregulation of slug and twist for activating mesenchymal characteristics in gastric cancer cells.^39^ On the other hand, suppression of SRPK1 by siRNA inhibited the expression of N-cadherin, snail and slug in glioblastoma and gastric cancer cells, particularly also decreasing the expression of matrix metalloproteinases (MMPs), and MMP2 and MMP9 proteins.^40-41^ This study presented the decreasing of MMP2 and MMP9 activities (Figure 4F). Furthermore, to suggest possible splicing downstream, many pro-metastatic spliced isoforms were mentioned, such as Rac1b,^42^ RONΔ165,^43^ AGR2vH,^44^ and the pro-angiogenic VEGF165^16^ which might be related to the metastatic progression of melanoma cells.

 In conclusion, this study demonstrated SRPK1 dysregulation in melanoma patients using dataset analysis, both the transcript abundance and correlation with the patients’ survival. Pan-SRPK inhibitor, SRPIN340 vs. SRPK1-specific inhibitor, and SPHINX31 were comparatively evaluated for their cellular effects in melanoma cells, A375 and MNT-1. SRPIN340 exhibited higher inhibitory effects on melanoma cell viability and growth. The inhibitors induced both melanoma cell apoptosis with downregulated Bcl-2 and upregulated cytochrome c proteins. They suppressed the invasive ability of A375 cells with downregulated vimentin mRNA and its transcription factors, snail. Particularly, SRPIN340 and SPHINX31 treatment altered vimentin protein expression and localization, as well as demonstrating lower MMP2 and MMP9 activity in A375 cells. Therefore, these data collectively suggest that SRPKs are a potential target for the development of antigrowth and antimetastatic substances for alternative melanoma treatments.

## Conclusion

 This study demonstrates that SRPK inhibitors can represent a potential therapeutic avenue in melanoma by modulating both cell survival and metastatic processes. SRPIN340 showed stronger activity than SPHINX31, reducing viability and growth, promoting apoptosis through Bcl-2 downregulation and cytochrome c release, and impairing invasion via altered vimentin expression and suppression of MMP2/MMP9 activity. These findings underscore the role of SRPKs in melanoma progression and provide mechanistic insights linking aberrant splicing regulation to cancer aggressiveness. Together, our findings provide preliminary evidence supporting the consideration of targeting splicing regulator SRPK as an alternative or complementary strategy for the development of melanoma therapies.

## Competing Interests

 None.

## Ethical Approval

 Not applicable.
